# Design and optimization of metamaterial-based highly-isolated MIMO antenna with high gain and beam tilting ability for 5G millimeter wave applications

**DOI:** 10.1038/s41598-024-53723-8

**Published:** 2024-02-08

**Authors:** Bashar A. F. Esmail, Slawomir Koziel

**Affiliations:** 1 Department of Enginering, Reykjavik University, 102 Reykjavík, Iceland; 2 Faculty of Electronics, Telecommunications and Informatics, Gdansk University of Technology, 80-233 Gdansk, Poland

**Keywords:** Engineering, Electrical and electronic engineering

## Abstract

This paper presents a wideband multiple-input multiple-output (MIMO) antenna with high gain and isolation, as well as beam tilting capability, for 5G millimeter wave (MMW) applications. A single bow-tie antenna fed by a substrate-integrated waveguide (SIW) is proposed to cover the 28 GHz band (26.5–29.5 GHz) with a maximum gain of 6.35 dB. To enhance the gain, H-shaped metamaterial (MM)-based components are incorporated into the antenna substrate. The trust-region (TR) gradient-based search algorithm is employed to optimize the H-shape dimensions and to achieve a maximum gain of 11.2 dB at 29.2 GHz. The MM structure offers zero index refraction at the desired range. Subsequently, the MIMO system is constructed with two vertically arranged radiators. Another MM, a modified square resonator (MSR), is embedded between the two radiators to reduce the mutual coupling and to tilt the antenna main beam. Herein, the TR algorithm is again used to optimize the MSR dimensions, and to enhance the isolation to a maximum of 75 dB at 28.6 GHz. Further, the MSR can tilt the E-plane radiation by ± 20° with respect to the end-fire direction when alternating between the two ports' excitation. The developed system is validated experimentally with a good matching between the simulated and measured data.

## Introduction

The emergence of fifth-generation (5G) technology is driven by the demand for broad bandwidth and high data rates to accommodate massive users. The millimeter-wave spectrum (MMW) offers an encouraging solution to enhance data rates and the capacity of 5G networks^1^. The International Telecommunications Union (ITU) has allocated various high-frequency bands to be associated with 5G, including 28, 38, and 60 GHz^2^. The 28 GHz (26.5–29.5 GHz) band is subject to extensive research for 5G networks, which exhibits significant performance enhancement over the 4G low frequencies. Nevertheless, the MMW band experiences high path losses, multipath fading effects, and interference as compared to sub-6 GHz bands^3^. High losses can be circumvented using high-gain antennas, whereas dual/multi-beam antennas are employed to conquer multipath fading and interference effects. The scientific literature reported a variety of techniques to enhance antenna gain, such as the use of multiple substrates^4^, the adoption of multiple shorting pins^5^, the incorporation of a dielectric lens^6^, and the utilization of artificial materials^7,8^. However, these approaches lead to bulky structures, complex power distribution, fabrication difficulties, narrow bandwidth, etc. It is evident that designing miniaturized high-gain antennas is a challenging task. Consequently, over the last two decades, metamaterials (MMs) have been utilized as a low-cost technique to enhance gain without significantly increasing the antenna profile. MMs constitute a remarkable class of synthetic materials that possess exceptional properties not found in nature. Various MM properties, including zero/near zero-index materials (ZIMs/NZIMs), low-index materials (LIMs), epsilon-near-zero (ENZ), mu-near-zero (MNZ), high refractive index (HRI), and negative refractive index (NRI), have been explored to design a broad range of antennas, especially in the context of gain enhancement^9^.

Improving the transmission quality in the MMW requires combining other available technologies, including MIMO, which has been identified as a key enabler of 5G. MIMO is a well-known diversity technique to enhance the resilience of communication when multiple antennas transmit/receive the same signal. This technique helps increase the channel capacity, spectrum efficiency, and data throughput, while reducing the impact of multipath effects^10,11^. Yet, a limited deployment volume of antenna systems may result in low isolation between MIMO elements, leading to system performance degradation. To address this, various techniques for reducing coupling have been suggested in the literature. These methods include introduction of decoupling networks and a neutralization line, as well as utilizing artificial materials^12,13^. Decoupling networks utilize explicit design procedures to systematically alleviate mutual coupling, whereas the neutralization line method is based on human intuition and lacks systematic procedures to improve isolation. Artificial materials with unique properties, such as electromagnetic band-gap (EBG) and metasurface, have been used as decoupling structures between radiating elements^14,15^. MMs can be embedded between MIMO radiators to mitigate the coupling without increasing the system's size and design complexity. Few reports utilized the MMs to improve the MIMO isolation at MMW^16–19^. The MM-based dielectric resonator antenna (DRA) was proposed to achieve high isolation of 29.3 dB and a gain of 7 dB at 28 GHz^16^. In^17^, a set of MMs was distributed between two microstrip antennas to improve the isolation by up to 40 dB and achieve a maximum gain of 8 dB at 30 GHz. Esmail et al.^18^ improved the dual-band MIMO's isolation using MMs, achieving a maximum of 47 dB at 38 GHz. However, these findings showed a low gain of only 5 dB at 38 GHz. In^19^, isolation of two-port MIMO at 60 GHz was enhanced to 35 dB by inserting a vertically positioned MM array between the two DRAs.

On the other hand, directing an antenna's beam in a predefined direction plays a crucial role in enhancing the communication system's performance in terms of quality of service, security, interference avoidance, and power conservation^20,21^. This property can be realized conventionally by means of the phased array antenna and Butler matrix networks. Nevertheless, these methods exhibit limitations, which include high-cost, complex transceiver systems, and high-profile structures. Moreover, most of these techniques suffer from a decrease in gain^22,23^. To overcome these issues, MMs are employed to achieve beam tilting while maintaining a compact system size and simplicity. Further, they can enhance the antenna performance figures, such as gain and efficiency^24,25^. With the prompt progress in wireless communications, there is a swelling demand for broadband MIMO antennas with high isolation and gain, as well as beam tilting properties at the MMW band to meet the needs of the 5G networks.

In the light of the mentioned literature, none of the stated MIMO systems discussed the employment of the optimization techniques to attain high isolation and gain, as well as beam tilting property at the MMW spectrum. On the other hand, application of rigorous optimization techniques is instrumental in successful handling of multiple geometry parameters and design objectives, which cannot be achieved through traditional means such as experience-driven parametric studies. This paper addresses a design and optimization of a high-performance MIMO antenna for 28 GHz 5G applications. Two sets of MMs are employed to improve the system performance. The first set uses an H-shaped resonator to enhance the gain of the single antenna, followed by the implementation of MIMO and integration with a modified square resonator (MSR) between the radiators to achieve high isolation and a deflected beam in the E-plane. Performance parameters of the system are enhanced through formal two-stage optimization of the two MM structures. The contributions of this work are summarized as follows:

I. Design a broad-bandwidth MMW antenna based on substrate-integrated waveguide (SIW) and integrate the H-shape MM to enhance the gain.
II. Implementation of a two-port MIMO antenna and embedding the MSR array between the radiators to exhibit high isolation of up to 75 dB at 28.6 GHz, as well as achieving the E-plane radiation tilting when changing between MIMO ports.
III. Development and execution of a customized two-stage optimization approach to enhance the gain of the individual bow-tie antenna, and to reduce the MIMO mutual coupling and maintain the deflection angles of ± 20° at 28 GHz. The TR gradient-based search algorithm is employed to optimize the geometrical dimensional of the two MMs, and to enhance the antenna and MIMO performance while maintaining a low profile and simplicity. For that purpose, a regularization-based objective function is defined, which enables a simultaneous control over the single antenna gain and its reflection response, as well as the MIMO isolation, beam tilting, and the reflection response.

Compared to the state-of-the-art developments reported in the literature, the proposed design provides a low-cost, low-profile, and lower-complexity system with high gain and isolation, as well as the capability of tilting the E-plane radiation. The remaining part of the work is organized as follows. Section “Antenna design” introduces the antenna design. Section “Gain enhancement based on optimized MMs” elaborates on gain enhancement involving MMs. Optimization of the antenna‘s MMs is discussed in Section “MMs antenna optimization”. Sections “MIMO design and isolation enhancement” and “MIMO performance optimization” present the MIMO implementation with improved performance based on the optimized MSR structure. Experimental results and discussions are provided in Section “Experimental results and discussion”. The paper is concluded in Section “Conclusion”.

## Antenna design

**Figure 1:**
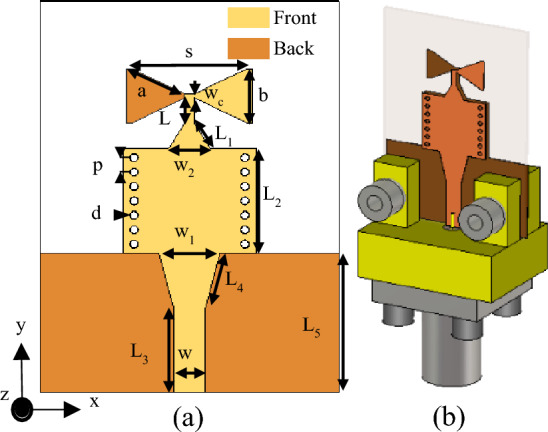
The configuration of the SIW-based antenna, (**a**) antenna geometry (The antenna dimensions are: *a* = 2.56, mm, *b* = 2.5 mm, *L* = 1.1 mm, *L*_1_ = 1.5 mm, *L*_*2*_ = 5 mm, *L*_3_ = 4 mm, *L*_4_ = 2.7 mm, *L*_5_ = 6.7 mm, *s* = 5.2 mm, *w*_*c*_ = 0.2 mm, *w* = 1.4 mm, *w*_1_ = 2.7 mm, *w*_2_ = 1.85 mm, *p* = 0.69 mm, *d* = 0.36 mm.), and (**b**) 3D view with the end-launch connector.

**Figure 2:**
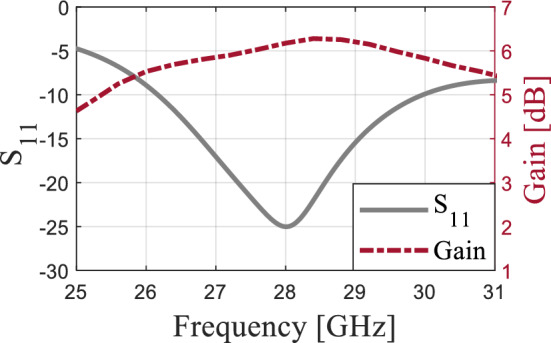
The antenna reflection coefficient and the realized gain.

The configuration of the proposed antenna has been shown in Fig. 1. It is essentially a bow-tie antenna fed by SIW. The structure consists of a microstrip line that feeds a microstrip-to-SIW transition, which, in turn, is connected to the front and the back parts of the bow-tie antenna. The antenna is printed on a Rogers RT5880 substrate with a thickness of 0.508 mm, dielectric constant of 2.2, and tangent loss of 0.0009. The SIW- based bow-tie antenna's dimensions are calculated using the set equations presented in^26^. The calculated values of *s*, *a*, *b*, and *w*_*c*_ are 4.9 mm, 2.4 mm, 2.33 mm, and 0.3 mm, respectively, which are used to obtain the initial design of the SIW-inspired bow-tie. Numerical simulations are then employed to fine-tune and finalize the antenna design, where the simulated values are *s* = 5.2 mm, *a* = 2.56 mm, *b* = 2.5 mm, and *w*_*c*_ = 0.2 mm. The presence of the SIW feed and inaccuracies in the design equations account for the minor discrepancy between the initial and final values. The SIW is employed to work as a wideband balun^27^. Both microstrip lines to the SIW and from the SIW to the two bow-tie arms are tapered to improve the impedance matching. Figure 2 depicts the antenna reflection coefficient and the gain. The results indicate that the antenna has a wide bandwidth of 3.8 GHz (26.2–30 GHz), making it suitable for covering the 5G band of 28 GHz. However, the maximum gain of 6.35 dB within the operational bandwidth is relatively low for MMW communications. There is a growing need for high-gain antennas to counteract the path loss experienced at the MMW spectrum. To address this, MMs have been integrated with antennas to boost their gain performance.

## Gain enhancement based on optimized MMs

**Figure 3:**
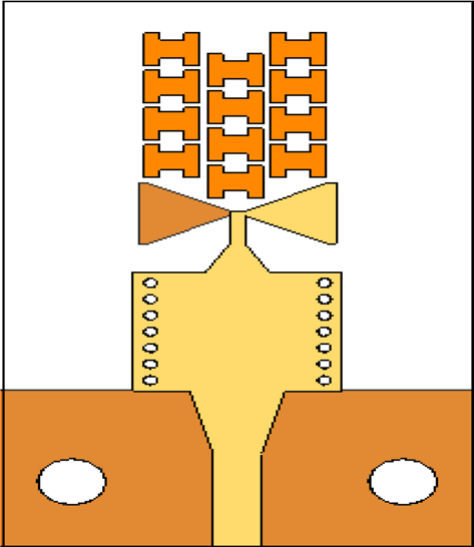
The configuration of the proposed antenna with the H-shaped MM structure.

**Figure 4:**
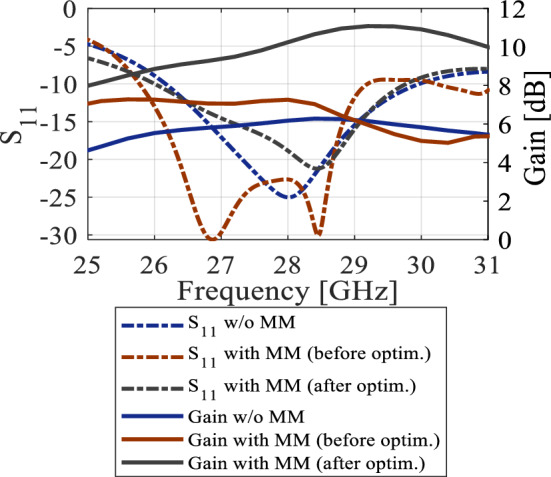
The reflection coefficients and gains of the antenna without and with the MSR structure (before and after the optimization).

An array of modified H-shaped MMs has been added to the antenna substrate in the end-fire direction (the *xy*-plane) to augment the gain. Figure 3 illustrates the antenna configuration with the MMs incorporated. By inserting the intial MMs into the antenna substrate and appropriate numerical optimization of their geometry parameters, it is possible to shorten the design cycle, including the adjustments to geometry dimensions, while achieving the optimum design. In this context, optimization of the MMs embedded into the antenna system (rather than designing the structure separately and then integrating it into the antenna substrate, which necessitates re-optimizing the number and location of unit cells), allows for saving time and resources, as compared to carrying out these stages separately. The reflection coefficient and gain plots of the stand-alone antenna and the MM antenna (before and after the optimization) have been depicted in Fig. 4. It can be observed that the inclusion of the MM has a minor impact on the impedance matching performance, where the bandwidth is only slightly shifted downwards to 26–29.8 GHz, compared to the initial antenna's bandwidth of 26.2–30 GHz. Nevertheless, the antenna remains capable of covering the 5G band (26.5–29.5 GHz). On the other hand, the stand-alone antenna's gain, which ranges from 5.8 to 6.35 dB in the 26.5–30 GHz band, is deemed insufficient for MMW communications. Before optimization, the initial H-shape design is incorporated in front of the radiation element. This preliminary design lacks the ZIM property, as indicated by the slight gain increase. As the gain of the antenna is known to be proportional to its aperture, the marginal gain increase before optimization can be attributed to the expansion of the substrate size to accommodate the MMs. The MM antenna's gain is notably enhanced prior to optimization, particularly below 29 GHz, compared to the stand-alone antenna. This suggests that further enhancements are achievable through suitable design optimization using formal numerical techniques. The TR gradient-based search procedure is employed to optimize the structure dimensions and to achieve a maximum gain of 11.2 dB at 29.2 GHz, cf. Figure 4. The details of the optimization process can be found in Section “MMs antenna optimization”.

## MMs antenna optimization

**Figure 5:**
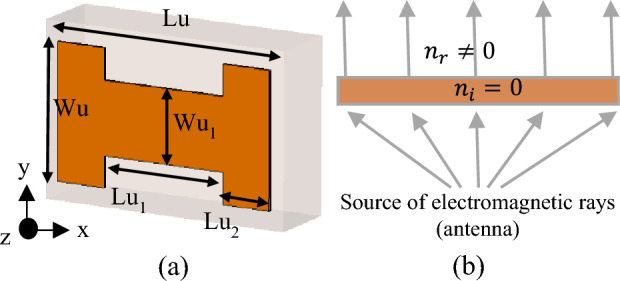
(**a**) Geometrical configuration of the optimized H-shaped resonator. (The dimensions are: *Lu* = 1.603 mm, *Wu* = 1.415 mm, *Lu*_1_ = (Lu-2* Lu_2_) = 0.699 mm, *Lu*_2_ = 0.452 mm, *Wu*_1_ = 0.755 mm), (**b**) the electromagnetic wave propagation based on the ZIM layer.

The proposed antenna is first optimized as a stand-alone (single) structure with the objective being gain enhancement. The adjustable parameters selected for the optimization process are ***x*** = [*Lu Wu Lu*_2_* Wu*_1_]^*T*^, all explained in Fig. 5a. The parameter space *X* is set up using the following ranges: 0.6 ≤ *Lu* ≤ 3 mm, 0.6 ≤ *Wu* ≤ 1.5 mm, 0.2 ≤ *Lu*_2_ ≤ 1 mm, and 0.2 ≤ *Wu*_1_ ≤ 1.6 mm. Furthermore, we have the following constraints, which are imposed to ensure the geometrical consistency of the antenna structure: *Lu*_2_ ≥ 0.2 mm, *Lu*_1_ = (*Lu − *2*Lu*_2_) ≥ 0.2 mm, and *Wu*_1_ ≤ *Wu − *0.4.

The primary objective is to increase the average in-band gain *G*(***x***) within the frequency range *F* = [26.5 29.5] GHz. The secondary objective is to ensure that |*S*_11_(***x***,*f*)| ≤ –10 dB for all *f* ∈ *F*. Consequently, the objective function (to be minimized) is defined as

1 $$U\left(x\right)=-G\left(x\right)+\beta {\left[\frac{{\text{max}}\{S\left(x\right)+\mathrm{10,0}\}}{10}\right]}^{2}$$

where

2 $$G\left(x\right)=\frac{1}{\left|F\right|} \underset{F}{\int }g(x,f)df$$

and

3 $$S\left(x\right)=\underset{\mathit{f }\in F}{{\text{max}}}\{|{S}_{11}(x,f)|\}$$

are the mean realized gain and maximum in-band reflection, respectively. The optimum design x^*^ is found by solving

4 $${{\varvec{x}}}^{*}={\text{arg}}\underset{\boldsymbol{x }\in {\varvec{X}}}{{\text{min}}}U({\varvec{x}})$$

**Figure 6:**
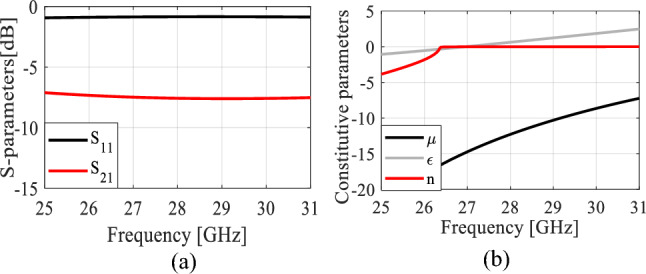
H-shaped resonator performance: (**a**) *S*-parameters and (**b**) the constitutive parameters.

**Figure 7:**
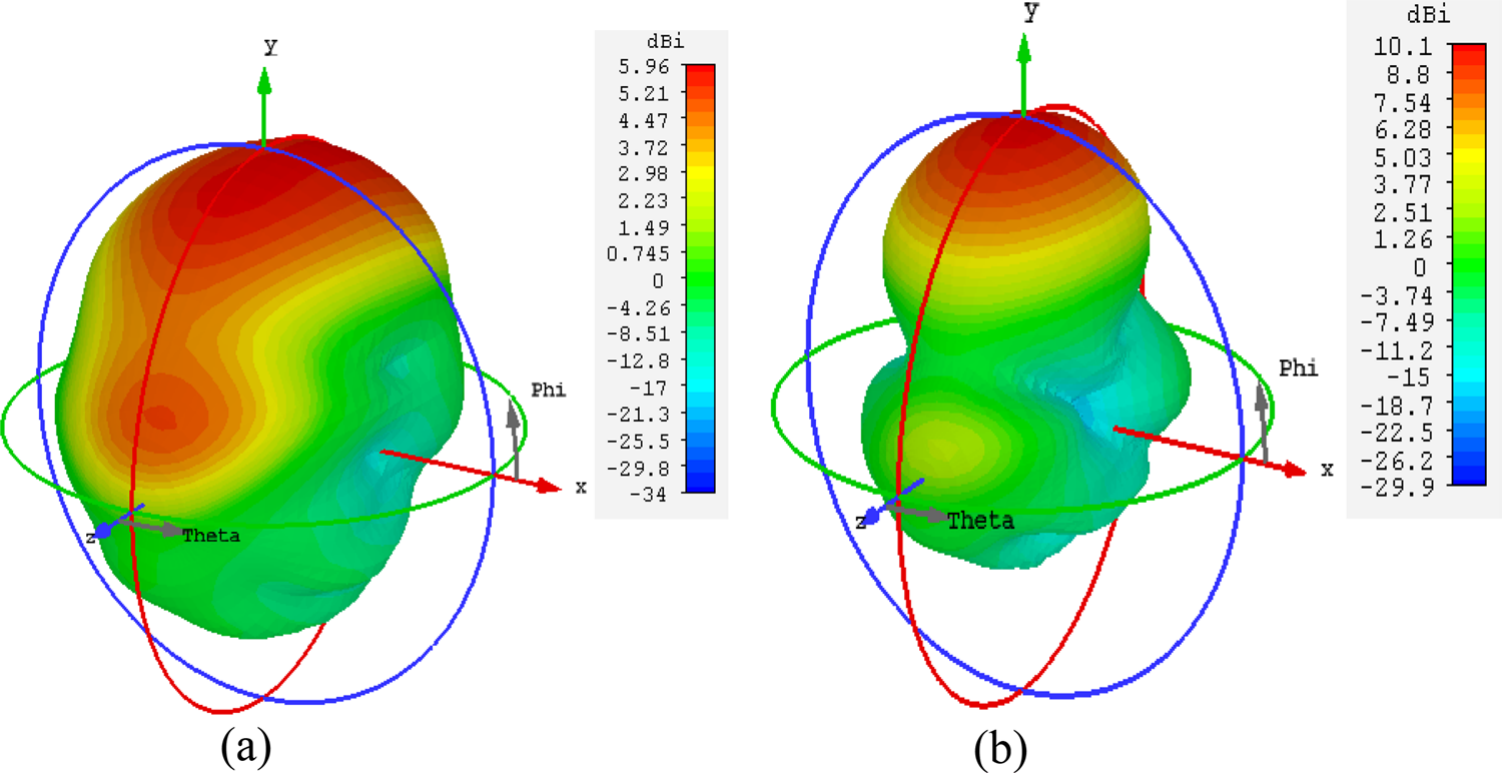
The 3D radiation patterns of the SIW-based antenna at 28 GHz, (**a**) without and (**b**) with ZIM.

Note that minimization of the objective function *U* leads to the improvement of the average gain, as well as enforcing the matching condition. The penalty coefficient *β*^28^ is set to 100 to ensure that |*S*_11_(***x***, *f*)| ≤ –10 dB over the bandwidth *F* within a tolerance of a fraction of dB. Formally speaking, the reflection requirement is an inequality constraint; however, due to being expensive to evaluate (required EM analysis), its implicit handling is more convenient^29^. The problem (4) is solved using the trust-region (TR) gradient-based algorithm^30^ with antenna response sensitivities estimated using finite differentiation^31^. The TR procedure yields a series ***x***^(*i*)^, *i* = 0, 1, …, of approximations to x^*^ as ***x***^(*i*+1)^ = argmin{***x***; ||***x*** – ***x***^(*i*)^|| ≤ *d*^(*i*)^: *U*_*L*_(***x***)}, where the local objective function *U*_*L*_ is defined as in (1) but evaluated using a first-order Taylor expansion model of antenna characteristics rather than directly through EM analysis. The search region size *d*^(*i*)^ is adaptively adjusted using conventional TR rules^30^. The TR sub-problem is solved using the SQP algorithm^32^ implemented in Matlab Optimization Toolbox^33^. The termination condition is convergence in argument (||***x***^(*i*+1)^ – ***x***^(*i*)^|| < *ε*; here, *ε* = 10^–3^). The initial design ***x***^(0)^ = [2 1.2 0.2 0.3]^*T*^ has been found using parametric studies. The optimized design obtained through optimization is x^*^ = [1.603 1.415 0.452 0.755]^*T*^ (dimensions in mm). The initial and optimized antenna responses can be found in Fig. 4. The optimized MM unit cell is shown in Fig. 5a. To ensure the required response, the boundary arrangement is assigned on all sides of the unit cell. The electric (magnetic) conductor boundary is allocated along the *x*(*z*)-direction. The *y*-axis is set to propagate a normal incident electromagnetic wave. Along the y-axis, the normally incident electromagnetic wave propagates. The incident waves excite the resonator structure, and electromagnetic interaction occurs within the unit cell, creating resonance in the transmitted and reflected waves. Choosing the y-axis for exciting the unit cell is justified by the fact that MIMO antennas serve as the origin of the electromagnetic wave, propagating through the unit cells along the y-direction—the established mode of propagation for the MM unit cell, as elucidated above. The structure response and the retrieved constitutive parameters are presented in Fig. 6a and b, respectively. The structure exhibits a zero-index characteristic within the desired range of 26.5–30 GHz, cf. Figure 6b. The zero-index metamaterial (ZIM) functions as a meta-lens, concentrating radiation in the emission direction. The 3D radiation patterns of the SIW-based antenna at 28 GHz without and with ZIM are depicted in Fig. 7a and b, respectively. The discernible change in the radiation pattern is evident in Fig. 7b, illustrating the meta-lens property of the MM. With ZIM loading, the antenna radiation displays heightened directivity in the end-fire direction along the y-axis, leading to a notable gain enhancement, as portrayed in Fig. 4. This remarkable feature, ZIM, can be leveraged to increase gain when combined with antennas. Gain enhancement can be explained using Snell's law of refraction, which is expressed as $sin\theta i\cdot {n}_{i }=sin\theta r\bullet {n}_{r },\mathrm{ where}$ *n*_*i*_(*θ*_*i*_) and *n*_*r*_(*θ*_*r*_) represent the refractive indices (angles) of the incident and refraction rays. Figure 5b depicts the wave propagation in both MM and air. Herein, when the incident rays pass from a medium of zero refractive index (*n*_*i*_ = *n*_*MM*_ = 0) to a high refractive index medium (*n*_*r*_ = *n*_*air*_ = 1), the refracted rays will disseminate in a path normal to the interface. Therefore, the phase change of the electromagnetic wave approaches/equals zero, leading to gain improvement in the emission direction.

## MIMO design and isolation enhancement

**Figure 8:**
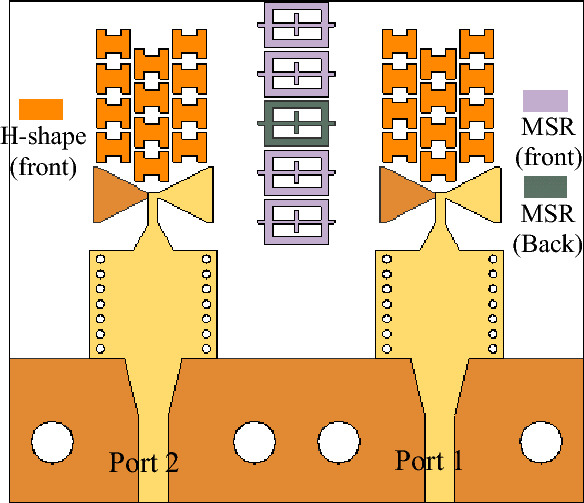
The configuration of the developed MM-based MIMO system with the MSRs. The configuration of the developed MM-based MIMO system with the MSRs.

**Figure 9:**
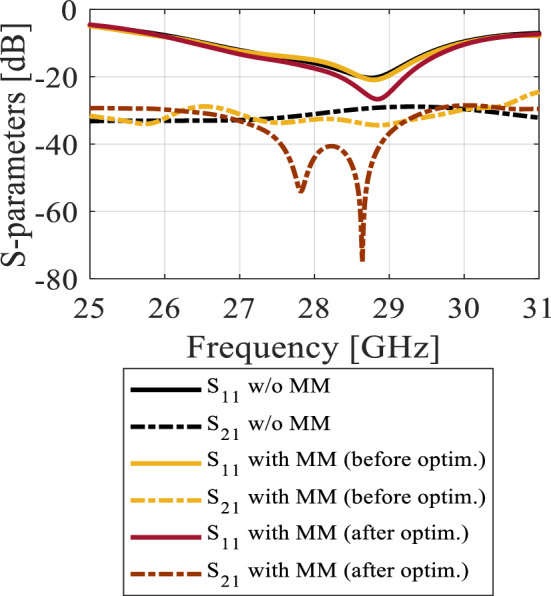
The S-parameters of the MIMO system without and with the MSR structure (before and after the optimization).

**Figure 10:**
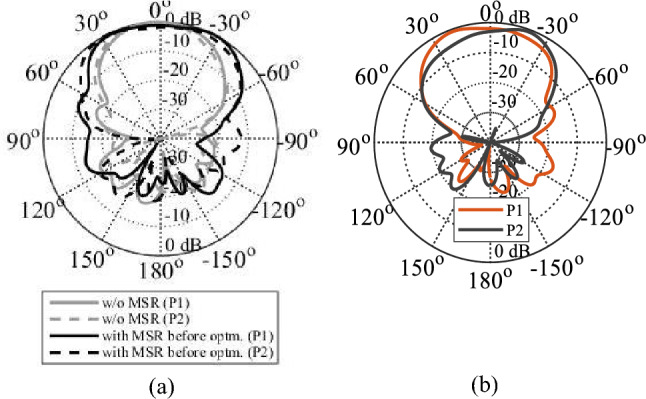
The E-plane radiation patterns of the MIMO system, (**a**) without and with MSR (before optimization), and (**b**) with MSR (after optimization).

As previously stated, the MIMO technology has been proposed to improve channel capacity, spectrum efficiency, and data throughput while reducing the multipath effects. To ensure optimal performance in such systems, minimizing mutual coupling is essential. Figure 8 illustrates the MM-based MIMO configuration, with radiators arranged adjacently. The reflection and transmission coefficients of the MIMO without MSRs are depicted in Fig. 9. The system provides isolation of better than 26 dB over the desired band. The performance is acceptable, but there is a room for isolation improvement, which would be beneficial, especially in dense MIMO scenarios. Figure 10a illustrates the E-plane radiation patterns for both ports, showing directive radiations towards the *y*-direction without any inclination. MMs offer a promising solution for minimizing mutual coupling compared to traditional methods. To boost isolation and to enable beam tilting in the end-fire direction (*y*-direction), an array of MSRs has been integrated between the two radiators, as depicted in Fig. 8. In most cases, achieving optimal performance in MM design hinges upon proper parameter tuning, and there exists no definitive formula that can be employed to design all MM structures. Consequently, utilization of formal optimization techniques represents the most efficient and effective means of obtaining desired outcomes in a shorter time frame and less effort.

## MIMO performance optimization

**Figure 11:**
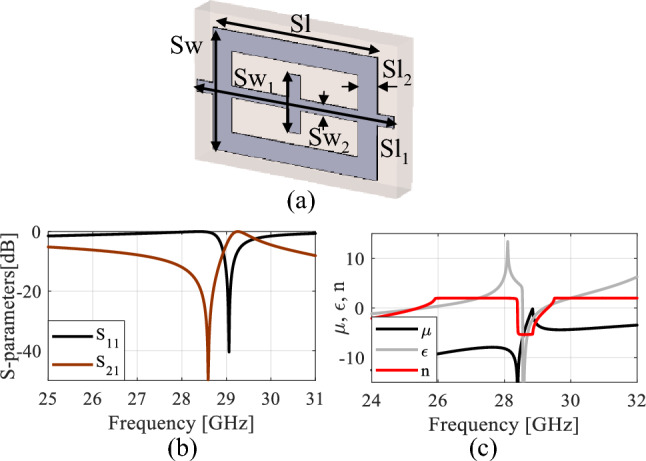
The MSR configuration and its performance, (**a**) the structure. (The dimensions are: *Sl* = 2.984 mm, *Sl*_1_ = 3.573 mm, *Sl*_2_ = 0.342 mm, *Sw* = 2.102 mm, *Sw*_1_ = 0.968 mm, *Sw*_2_ = 0.203 mm), (**b**) *S*-parameters, and (**c**) the constitutive parameters.

The MIMO version of the proposed antenna is optimized to ensure the required tilt of the E-plane radiation pattern (here, *α*_0_ = 20°) at 28 GHz, as well as to improve antenna isolation |*S*_21_| within the frequency range *F* = [26.5 29.5] GHz. Additional conditions are to maintain sufficient impedance matching |*S*_11_(***x***,*f*)| ≤ –10 dB, and gain *g*(***x***,*f*) ≥ 9 dB for all *f* ∈ *F*. The design variables selected for the optimization purpose are ***x*** = [*Sl Sw Sl*_2_ *Sw*_1_ *Sw*_2_ *Sl*_1_]^*T*^*,* all explained in Fig. 11a. The parameter space *X* is set using the following ranges: 1 ≤ *Sl* ≤ 4 mm, 1.4 ≤ *Sw* ≤ 2.3 mm, 0.2 ≤ *Sl*_*2*_ ≤ 0.5 mm, 0.7 ≤ *Sw*_1_ ≤ 1.3 mm, 0.2 ≤ *Sw*_2_ ≤ 0.4 mm, and 2.7 ≤ *Sl*_1_ ≤ 4.5 mm. Additionally, we have three geometry constraints: *Sw*_1_ ≤ ((*Sw* − 2*Sl*_2_) − 0.4), *Sl*_1_ ≥ *Sl*, and *Sw*_2_ ≤ *Sw*_1_ − 0.4. The primary objective is to reduce the antenna isolation, defined as *I*(***x***) = max{*f* ∈ *F*: |*S*_21_(***x***,*f*)|} (i.e., as the maximum in-band transmission). The remaining conditions are handled as constraints. The objective function to be minimized is therefore defined as

5 $${U}_{1}\left(x\right)=I\left(x\right)+{\beta }_{1}{\left[\frac{{\text{max}}\{S\left(x\right)+\mathrm{10,0}\}}{10}\right]}^{2}+{ \beta }_{2}{\left[\frac{{\text{max}}\{{G}_{m}\left(x\right)-\mathrm{9,0}\}}{9}\right]}^{2}+{\beta }_{3}{\left[\alpha \left(x\right)-{\alpha }_{0}\right]}^{2}$$

where *S*(***x***) is defined in (3), the minimum in-band gain is defined as

6 $${G}_{m}\left(x\right)=\underset{\mathit{f }\in F}{{\text{max}}}\{g\left(x,f\right)\}$$

whereas *α*(***x***) is the angle of maximum directivity extracted from the EM-simulated antenna farfield. The first two constraints are of inequality type with their violations quantified as relative ones, whereas the last one is the equality condition. The penalty coefficients are set to *β*_1_ = *β*_2_ = *β*_3_ = 100. It should be noted that although matching and gain conditions are formally included into the objective function, they are to be almost automatically satisfied as the reflection coefficient and gain are only weakly-dependent on the parameters selected for isolation improvement. The initial design, obtained through parametric studies, is ***x***^(0)^ = [3.5 2.25 0.32 0.6 0.4 3.4]^*T*^ [mm]. The optimized design x^*^ = [2.984 2.102 0.342 0.968 0.203 3.573]^*T*^ has been found by minimizing the objective function (5) similarly as in (4), using the trust-region algorithm outlined before. The initial and optimized antenna responses can be found in Figs. 8 and 9.

**Figure 12:**
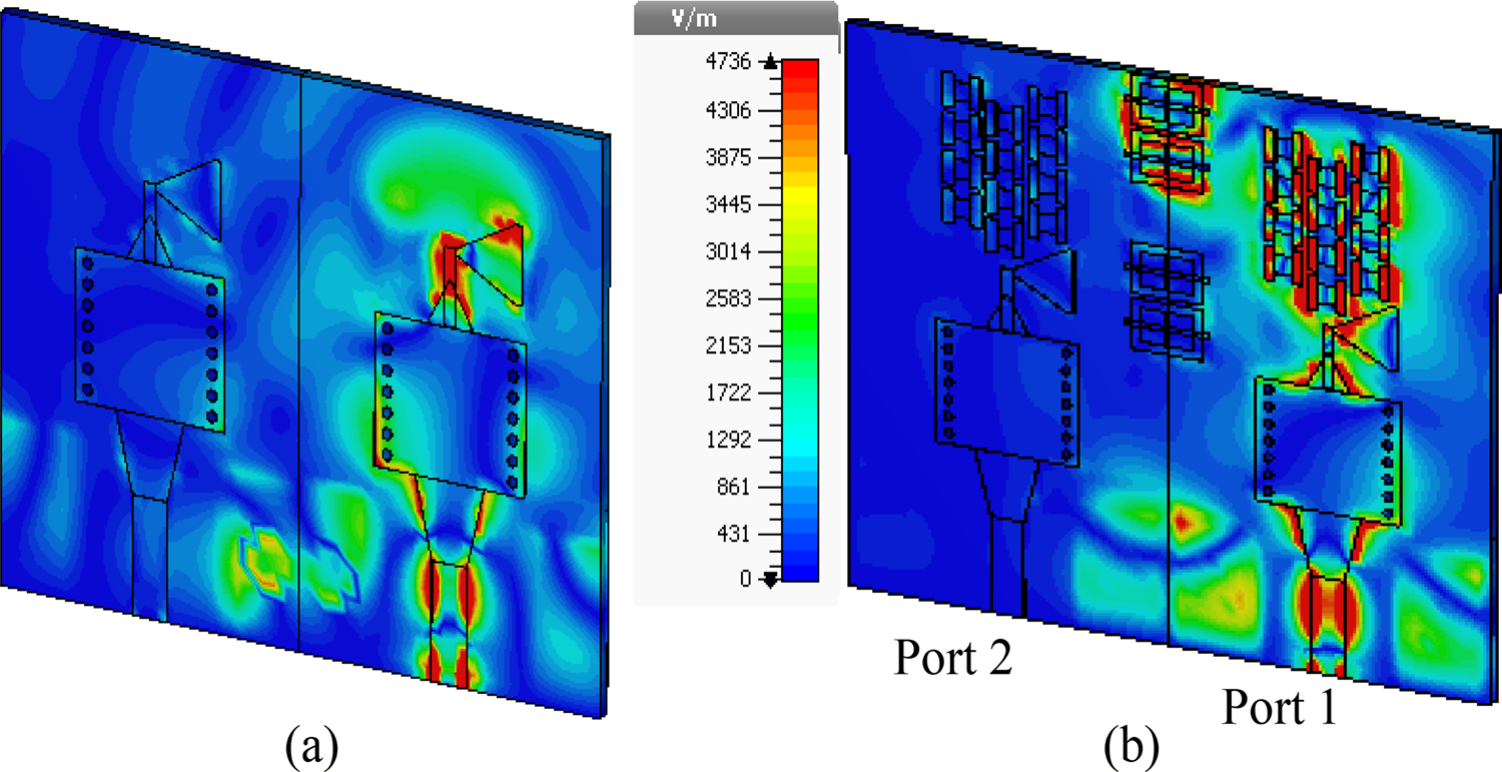
Electric field (V/m) distributions of the MIMO system at 28.6 GHz (**a**) without MMs and (**b**) with MMs.

Figure 11a illustrates the MSR unit cell configuration, where the boundaries are established similarly to that of the H-shaped resonator. The structure consists of a square ring and two cross bars. The response of the structure and its constitutive parameters are presented in Fig. 11b and c, respectively. Notably, the structure exhibits negative values of *ɛ*, *μ*, and *n* at the resonance frequency of 28.6 GHz. The optimized MSR offers high isolation in the desired band with a maximum of 75 dB at 28.6 GHz, with an enhancement of about 47 dB over the bare MIMO system, cf. Figure 9. Further, the structure demonstrates a substantial permittivity of 6.5 and a refractive index of 2 at 28 GHz. It is worth noting that the actual substrate's refractive index is merely 1.6^34^. Hence, the proposed MM exhibits a significantly higher refractive index than the substrate, creating two different media with different refractive indices in the proximity of the radiator. This configuration causes the antenna beam to tilt toward the medium of a higher refractive index (MSR configuration). Excitation of Port 1 results in a + 20° tilt in the E-plane, whereas exciting Port 2 produces a deflection angle of − 20°, cf. Figure 10b. The electric field distributions at 28.6 GHz for the MIMO system without and with MMs are presented in Fig. 12a and b, where Port 1 is excited, and Port 2 is terminated by a 50-Ω load. During the simulation of the first antenna (Port 1) without the MMs, a significant mutual coupling field is evident at the second antenna (Port 2), as illustrated in Fig. 12a. Conversely, with the inclusion of MMs, the coupling field diminishes at the second antenna, as shown in Fig. 12b, resulting in a reduction of mutual coupling. The upper MSR unit cells play a more substantial role in minimizing mutual coupling. It's worth noting that the gain enhancement is substantiated by contrasting the electric fields of the bare MIMO and MM-based MIMO. The improvement in gain is evident as the field is distributed through the ZIMs, as shown in Fig. 12b.

## Experimental results and discussion

**Figure 13:**
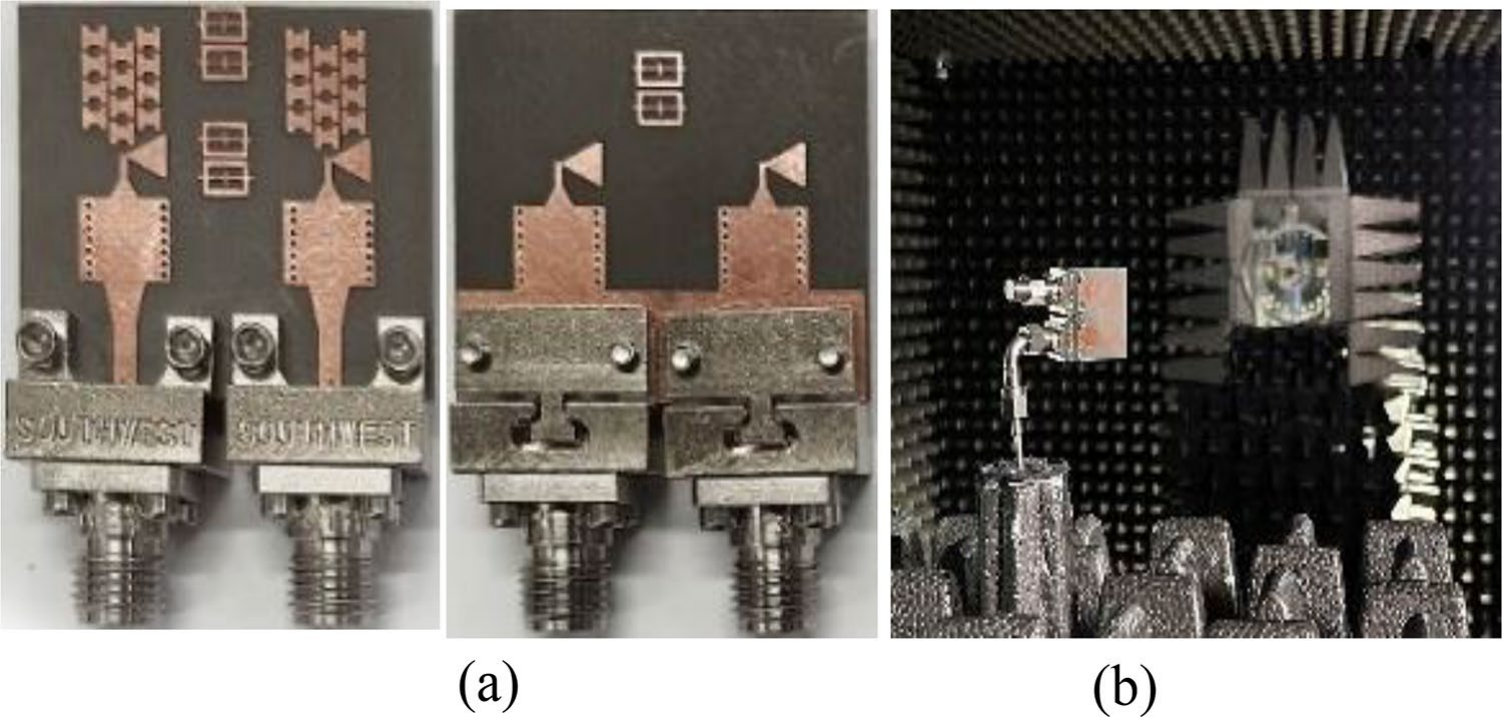
Fabrication and measurement: (**a**) front and back views of the MIMO prototype and (**b**) the MIMO antenna in the anechoic chamber.

**Figure 14:**
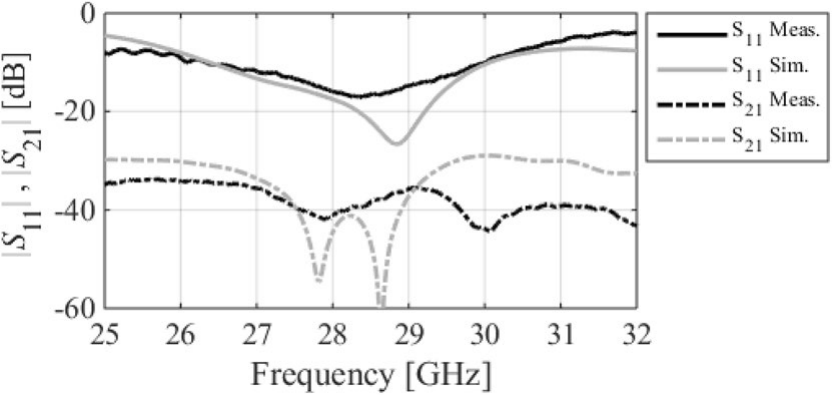
Simulated and measured reflection and transmission coefficients.

**Figure 15:**
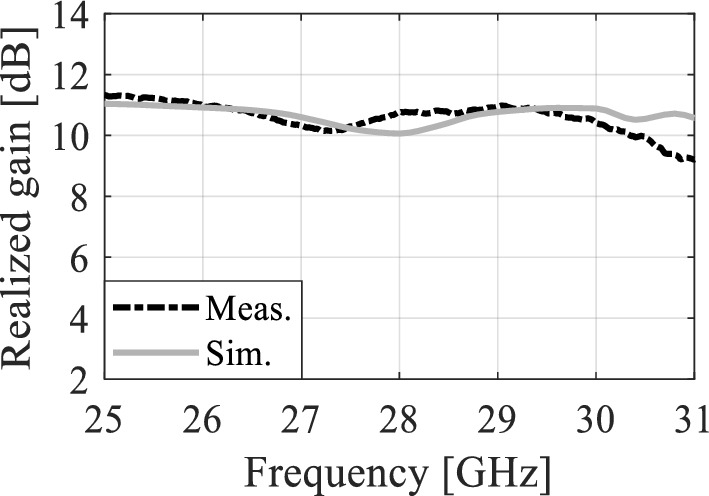
Simulated and measured gain.

**Figure 16:**
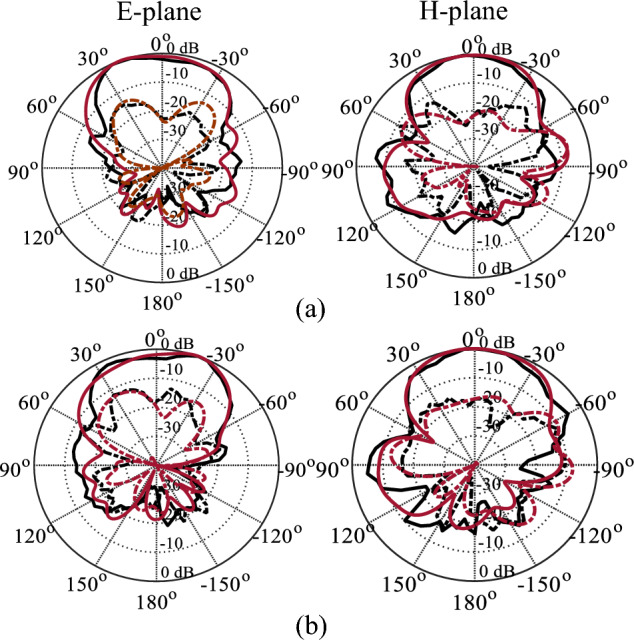
Simulated and measured radiation patterns of the developed MIMO system in the E- and H-planes at 28 GHz, (**a**) Port 1 and (**b**) Port 2.

**Figure 17:**
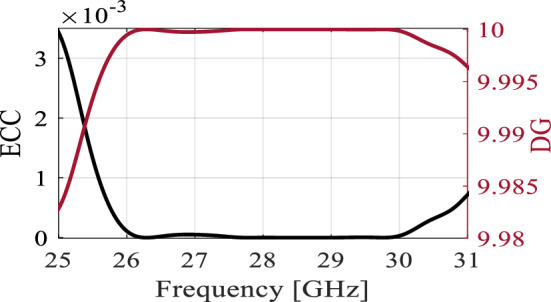
The diversity performance of MIMO system, ECC and DG.

The optimized MM-based MIMO has been fabricated to corroborate the numerical simulation outcomes and showcase its relevance for 5G applications. Figure 13a displays the front and back views of the MIMO antenna prototype. An Anritsu vector network analyzer MS4644B (0–40 GHz) was utilized to measure the reflection and transmission coefficients. The radiation patterns were validated in an anechoic chamber, as depicted in Fig. 13b. Figure 14 illustrates the comparison between the simulated and measured |*S*_11_| and mutual coupling |*S*_21_|. The developed MIMO provides a wide impedance bandwidth ranging from 26.2 to 30 GHz, effectively covering the 5G band of 28 GHz (26.5–29.5 GHz). One can observe a satisfactory alignment between both datasets for |*S*_11_|. Nonetheless, a slight difference in resonant magnitude is observed between the measured and simulated data. Measured isolation, |*S*_21_|, indicates a good agreement with simulation, especially in frequencies below 28 GHz. The measured isolation results, particularly above 28 GHz, are notably inconsistent with the simulated data due to various factors, including fabrication tolerance, cable loss, assembly inaccuracies, and the use of bulky and closely spaced end-launch connectors. Figure 15 displays the MM-based MIMO's simulated and measured realized gain plots when Port 1 is excited, and Port 2 is terminated with a 50-Ω load. In the desired band of 26.5–29.5 GHz, the simulated realized gain varies from 10 to 11 dB. The measured gain matches the simulation result for the entire band, although a minor discrepancy is observed at 28 GHz. This discrepancy is likely due to the same reasons mentioned earlier, such as manufacturing and antenna assembly inaccuracies, as well as angular inaccuracy in the antenna placement in the chamber. The co- and cross-polarization radiation patterns of the MM-inspired MIMO system in the E- and H-planes at 28 GHz are displayed in Fig. 16. The radiation results are introduced individually for Port 1 and Port 2, cf. Figure 16a and b, respectively. The developed system offers directional radiation in both planes with a low level of cross-polarized fields. Based on the ports excitation, the measured results confirm that the E-plane radiation is tilted by ± 20° at 28 GHz. The measured radiations correspond well with numerical simulation results for the E and H-planes at 28 GHz. Notably, the inactive port is terminated using the 50-Ω matched load during the measurements. The MM-based MIMO is investigated in terms of the envelope correlation coefficient (ECC) and diversity gain (DG) to appraise the system performance. The ECC can be computed using the field-based formula and directly calculate DG from the resulting ECC^17^. Figure 17 shows the ECC and DG plots. The ECC is < 0.5∙10^–4^ in the range of 26.5–29.5 GHz, which is considerably below the acceptable threshold of 0.5 for wireless systems. Additionally, the DG values are in proximity to the standard value of 10 dB.

Table 1 illustrates a performance comparison between the proposed MM-based MIMO and state-of-the-art structures reported in the recent literature. In contrast to the recent findings, the MIMO antenna developed in this work exhibits the highest isolation and gain. Further, it boasts a compact and low-profile design, a broad bandwidth, and exceptional diversity performance while also providing a beam tilting capability.

**Table 1:** Comparison of the developed MIMO system with state-of-the-art structures.

Ref.	MIMO antenna type (2D/3D)	Frequency (bandwidth) GHz	Isolation (max. dB)	Gain (dB)	Beam tilting	ECC	DG (dB)
^16^	DRA (3D)	28 (26.6–29.5)	29.34	7	No	0.005	9.96
^17^	Microstrip antenna (2D)	30 (29–31)	40 dB	8	No	0.01	9.98
^18^	Monopole antenna (2D)	28 (27.1–28.8), 38 (36–39)	34.6 at 28 and 37 at 38 GHz	6.6 at 28 and 5 at 38 GHz	No	0.2 × 10^–4^	9.99
^19^	DRA (3D)	60 (57–62)	35	–	No	0.1 × 10^–4^	–
This work	SIW-based bow tie (2D)	28.6 GHz (26.2–30)	75	11	Yes ± 20°	0.5 × 10^–4^	9.999

## Conclusion

The MM-based MIMO on single-layer Rogers PCB with broad bandwidth, high isolation and gain, and beam tilting capability in E-plane is reported for 5G MMW applications. The proposed antenna is a bow-tie structure fed by a SIW, covering a 5G band of 28 GHz, with H-shaped resonators integrated into the substrate to augment the gain. The H-shape dimensions are optimized using the TR algorithm to achieve zero index refraction within the desired range, thereby a maximum gain of 11.2 dB at 29.2 GHz. The MIMO system is then constructed using two vertically arranged radiators, with a modified square resonator (MSR) MM embedded between the radiators to reduce mutual coupling and to achieve beam tilting. The TR algorithm is employed to optimize the MSR dimensions and achieve a maximum isolation of 75 dB at 28.6 GHz, as well as E-plane radiation tilting of ± 20° in the end-fire direction when switching between the two ports. The MIMO system is experimentally validated, with a good matching between the simulated and measured data. Overall, this design offers a low-cost, low-profile, and less complex system with high isolation and gain as well as beam tilting capability compared to state-of-the-art developments in reported the literature. We believe that this design has the potential to be expanded to various antennas incorporating a range of MMs, allowing for the attainment of high-deflection angles in both the E- and H-planes. Furthermore, the system could be scaled to support multiple ports, beyond the current configuration of two, to further enhance 5G channel capacity.

## Data Availability

All data has been included in study.
